# Non-canonical LexA proteins regulate the SOS response in the Bacteroidetes

**DOI:** 10.1093/nar/gkab773

**Published:** 2021-10-06

**Authors:** Miquel Sánchez-Osuna, Pilar Cortés, Mark Lee, Aaron T Smith, Jordi Barbé, Ivan Erill

**Affiliations:** Departament de Genètica i de Microbiologia, Universitat Autònoma de Barcelona, 08192 Bellaterra, Spain; Departament de Genètica i de Microbiologia, Universitat Autònoma de Barcelona, 08192 Bellaterra, Spain; Department of Chemistry and Biochemistry, University of Maryland Baltimore County, Baltimore, MD 21250, USA; Department of Chemistry and Biochemistry, University of Maryland Baltimore County, Baltimore, MD 21250, USA; Departament de Genètica i de Microbiologia, Universitat Autònoma de Barcelona, 08192 Bellaterra, Spain; Departament de Genètica i de Microbiologia, Universitat Autònoma de Barcelona, 08192 Bellaterra, Spain; Department of Biological Sciences, University of Maryland Baltimore County, Baltimore, MD 21250, USA

## Abstract

Lesions to DNA compromise chromosome integrity, posing a direct threat to cell survival. The bacterial SOS response is a widespread transcriptional regulatory mechanism to address DNA damage. This response is coordinated by the LexA transcriptional repressor, which controls genes involved in DNA repair, mutagenesis and cell-cycle control. To date, the SOS response has been characterized in most major bacterial groups, with the notable exception of the Bacteroidetes. No LexA homologs had been identified in this large, diverse and ecologically important phylum, suggesting that it lacked an inducible mechanism to address DNA damage. Here, we report the identification of a novel family of transcriptional repressors in the Bacteroidetes that orchestrate a canonical response to DNA damage in this phylum. These proteins belong to the S24 peptidase family, but are structurally different from LexA. Their N-terminal domain is most closely related to CI-type bacteriophage repressors, suggesting that they may have originated from phage lytic phase repressors. Given their role as SOS regulators, however, we propose to designate them as non-canonical LexA proteins. The identification of a new class of repressors orchestrating the SOS response illuminates long-standing questions regarding the origin and plasticity of this transcriptional network.

## INTRODUCTION

Many environmental insults and endogenous processes can cause DNA lesions that pose a direct threat to cell survival. In most bacterial species, DNA damage is addressed by a transcriptional regulatory process known as the SOS response (1,2). First described in *Escherichia coli*, the SOS response involves the coordinated expression of over 40 genes encoding primarily DNA repair and recombination enzymes, translesion synthesis polymerases and cell division inhibitors (3,4). This process is governed by the LexA repressor. This protein forms dimers that bind to operator sites in the promoter region of regulated operons by targeting a highly specific palindromic motif (CTGT-N8-ACAG in *E. coli*) (3). Following DNA damage, single-stranded DNA (ssDNA) fragments stemming from stalled replication forks are bound by the recombination protein RecA, resulting in active nucleoprotein filaments that are capable of inducing the autocatalytic cleavage of the LexA protein (5,6). Upon self-cleavage, the LexA dimer detaches from its operator sites, derepressing regulated operons and inducing the SOS response (4).

Over the last three decades, the SOS response has been documented in multiple bacterial groups (1,2,7–10). This broad taxonomic perspective has revealed a minimal set of core genes that are predominantly regulated by LexA across phyla. These encompass the *lexA* and *recA* genes, as well as those encoding the type IV (*dinB*) and type V (*umuDC*) error-prone DNA polymerases (1). The systematic analysis of this system has also uncovered that, in contrast with many other transcriptional regulators, the LexA repressor has significantly changed its binding motif through evolution. Reported LexA-binding motifs range from short (TTAC-N3-GTAA; *Bdellovibrio bacteriovorus*) to large palindromes (GGTT-N10-AACC; *Geobacter sulfurreducens*) and include several direct-repeat motifs (GTTC-N7-GTTC; Alphaproteobacteria) (1). Duplications of the *lexA* gene are often associated with LexA-binding motif divergence, enabling the redundant *lexA* gene to significantly alter its binding motif (11). It has been postulated that, upon loss of the primary *lexA* gene, the diverged LexA protein will gradually retake control of the SOS regulatory network via a process of convergent evolution (7).

LexA monomers contain an N-terminal winged helix–turn–helix (wHTH) DNA-binding domain with three α helices (PF01726) and a C-terminal autolysis and dimerization domain (PF00717). These two domains are connected by a flexible linker region (12). Several other proteins share the S24 serine peptidase family (PF00717) catalytic C-terminal domain of LexA. The *Enterobacteria* phage Lambda CI and *Salmonella* phage P22 C2 repressors bind DNA via an N-terminal HTH domain encompassing five alpha helices and repress genes that participate in the lytic cycle of temperate bacteriophages. Like LexA, CI and C2 repressors are capable of undergoing self-catalytic cleavage via interaction with RecA nucleoprotein filaments, triggering the phage lytic cycle (13). The *E. coli* UmuD protein forms part of the DNA polymerase V complex with UmuC. RecA-mediated self-catalytic cleavage of UmuD activates the UmuDC complex, providing a post-translational level of control on this highly mutagenic polymerase in addition to its transcriptional regulation by LexA (14,15).

Given its importance in maintaining cell viability, the SOS response is often assumed to be universal. The absence of LexA homologs, however, has been reported in several bacterial groups. Beyond endosymbiotic bacteria that have undergone substantial genomic reduction, such as the *Rickettsiae* (16), the absence of LexA homologs has been particularly well documented in the *Streptococcaceae*. In the absence of LexA, different *Streptococcaceae* species have been shown to orchestrate a basic response to DNA damage using a LexA-like repressor, HdiR. In *Lactococcus lactis*, where it was first reported, HdiR was shown to regulate itself and a UmuC homolog in response to DNA damage (17). As is the case for LexA and phage repressors, RecA nucleoprotein filaments trigger self-catalytic cleavage of HdiR, but further processing of HdiR by the Clp proteolytic complex is required for full derepression (17). Subsequent work revealed basic SOS-like regulatory networks under control of HdiR in *Streptococcus* species. In *Streptococcus uberis*, HdiR regulates only a mutagenic gene cassette, while in *Streptococcus thermophilus*, it regulates both the *cinA-recA* operon and two *umuC* loci (18,19). Interestingly, non-SOS regulatory functions have also been reported for HdiR in the *Streptococcaceae*. In *L. lactis*, HdiR was shown to respond to heat shock, whereas in *S. thermophilus* HdiR was found to interfere with natural transformation.

The uptake of a minimal SOS-like network by proteins other than LexA has also been reported in the *Moraxellaceae*, a bacterial family with no known LexA homologs. In *Acinetobacter baumannii* and *Acinetobacter baylyi*, a variant of the UmuD protein containing an N-terminal DNA-binding domain (UmuDAb) has been shown to regulate the expression of its own and several other *umuDC* operons (20,21). Like LexA and HdiR, UmuDAb undergoes autocatalytic cleavage upon interaction with RecA nucleoprotein filaments, but its regulatory function appears to be restricted to the control of type V error-prone polymerases (21,22).

The Bacteroidetes phylum comprises a large and diverse group of bacteria present in many different ecosystems, such as freshwater and marine habitats, soil, plants and the mammalian gastrointestinal tract, and ranging from temperate to tropical and polar climates (23). In all these ecosystems, the Bacteroidetes play important roles, and are essential for the degradation of complex carbohydrate-based biomass (24). Due to the wide variety of habitats they occupy, members of the Bacteroidetes phylum are frequently exposed to many DNA damaging agents including antibiotics and reactive oxygen species (ROS). However, the presence of a DNA repair system similar to the SOS network has not been reported to date in this bacterial phylum. The sequencing of the first representative Bacteroidetes complete genomes (*Bacteroides thetaiotaomicron* and *Bacteroides fragilis*) exposed the lack of LexA homologs in these bacterial species, suggesting that this phylum lacked a conventional SOS response (1). The recent description of a conventional LexA regulon in the Bacteroidetes sister phylum Balneolaeota prompted us to investigate the SOS response in the Bacteroidetes (10).

In this work we combine comparative genomics approaches with *in vitro* and *in vivo* analyses to elucidate the SOS response in the Bacteroidetes. We report that a novel family of LexA-like repressors, targeting two distinct palindromic motifs, controls a conventional SOS response in this phylum. Our results highlight the evolutionary plasticity of this transcriptional network and support the notion that the SOS response has reevolved several times through convergent evolution using S24-family peptidases with distinct DNA-binding domains.

## MATERIALS AND METHODS

### Genome data and ortholog detection

Bacteroidetes complete genome assemblies were downloaded from the RefSeq database (25) both in protein multi-FASTA and nucleotide GenBank formats. Orthologs for UmuD and DinB/UmuC were identified in all the Bacteroidetes RefSeq complete genome assemblies using HMMER (*hmmsearch*, E-value 1^e-10^) (26), using Hidden Markov Models (HMM) derived for the Clusters of Orthologous Groups (COGs) mapping to *E. coli* UmuD (COG1974) and DinB/UmuC (COG0389) as queries (27,28). Orthologs for the Bacteroidetes RecA protein and for the two putative Bacteroidetes SOS regulators were obtained by searching all available Bacteroidetes complete proteomes with BLASTP (limiting e-value 1e^–20^ and query coverage > 75%), using as queries all the instances of these proteins identified in the comparative genomics analyses, and subsequently removing duplicates.

### Motif discovery and comparative genomics analysis

The upstream regions (from −250 to +2 bp of the predicted translational start site (TLS)) of identified UmuD and DinB/UmuC orthologs, and subsequently for putative Bacteroidetes SOS regulators, were obtained from the respective complete genome sequences. Redundant upstream sequences (those with nucleotide sequence identity > 75 %) were removed, and the resulting non-redundant panel was used to perform motif discovery. Motifs were inferred with MEME (29) using a 12–26 bp motif size, the Any Number of Repetitions (ANR) site distribution model and otherwise default parameters. Refined motif discovery was performed by activating the *-pal* option in MEME to restrict the search to palindromic motifs and otherwise identical parameters. Comparative genomics analyses of the regulatory networks defined by the identified motifs were performed using the CGB comparative genomics platform, using COG and PFAM for functional annotation of orthologous groups (10). CGB configuration files for regulon reconstruction are provided as supplementary material in JSON format (Supplementary Data S1, Supplementary Data S2).

### Protein sequence and *in silico* structural analyses

A multiple sequence alignment of the C-terminal region of the putative Bacteroidetes SOS regulators and known S24 catalytic domains was performed with CLUSTALW in profile alignment mode, using the *E. coli* LexA protein structure (P0A7C2) to define a gap penalty mask (30). Consensus sequences for each group of putative Bacteroidetes SOS regulators were inferred with the EMBOSS Cons service (31), and the multiple sequence alignment was plotted with BioEdit (32). HMM for the two clusters of putative Bacteroidetes SOS regulators were obtained by performing independent multiple sequence alignments of each cluster of sequences with CLUSTALW, and generating the HMM with the *hmmbuild* command of the HMMER suite.

Experimentally-determined structures of *E. coli* LexA (PDB ID: 3JSO), full-length *Enterobacteria* phage Lambda (PDB ID: 3BDN) and the C-terminal domain of Lambda Cl (PDB ID: 1F39) were obtained from the Protein Databank (PDB). Calculated structures were determined via the Robetta suite (33,34) using the TrRosetta, comparative modelling, and *ab initio* strategies independently. All three strategies demonstrated a qualitative similarity regardless of modelling pipeline. After observation of all outputs, TrRosetta produced models with the highest confidence interval across the entirety of the polypeptide and was therefore used for comparative analysis against the experimentally-determined structures. Structural superpositions and Cα root-mean-square deviations (RMSDs) were calculated using PyMol (35) after manual trimming to remove unstructured regions belonging to the flexible linker connecting the N- and C-terminal domains. The numbers of Cαs used for each pairwise RMSD calculation are indicated in the figure legend.

### Phylogenetic inference

For phylogenetic inference of putative SOS regulators, the amino acid sequences of all putative SOS regulators identified in the comparative genomics analyses were combined with all the representative sequences for COG1974 and COG2932 available in the COG database (27). For RecA, records for one representative member of each Bacteroidetes family, as well as for all the species used in motif discovery, were selected for phylogenetic inference. In both cases, a protein sequence multiple sequence alignment was generated using T-COFFEE (36), combining three CLUSTALW amino acid sequence alignments with different (5,10,25) gap opening penalties and a single T-COFFEE *Lalign* method amino acid sequence alignment (37). For RecA, the resulting amino acid sequence alignment was processed with Gblocks using the half-gap setting (38). Phylogenetic inference was carried out with MrBayes (39). Four Metropolis-coupled Markov chain Monte Carlo simulations with four independent chains were run for 5 000 000 (SOS regulators) and 10 000 000 (RecA) generations, using a mixed four-category gamma distributed rate plus proportion of invariable sites model [invgamma] and a JTT (Jones–Taylor–Thornton) amino acid substitution model (40). Convergence was monitored with Tracer (41), imposing the restriction that the estimated sample size (ESS) be above 200 and that the potential scale reduction factor (PSRF) be within 0.005 of 1, and burn-in was set at 25% of iterations. A consensus tree was generated with the *all-compat* option. Tree visualization and annotation were performed with iTOL (42).

### Protein purification and electro-mobility shift assays

The *Salegentibacter agarivorans* [BM084_RS03395], *Pontibacter actiniarum* [CA264_RS07585] and *Elizabethkingia anophelis* [BBD30_RS12205] genes encoding putative Bacteroidetes SOS regulators (WP_075324850.1, WP_025606010.1 and WP_078407279.1, respectively) were synthesized by ATG:biosyntheticsGmbH, Germany, and cloned into a dephosphorylated pUA1108 vector (43) using an NdeI and BamHI (New England Biolabs) double digest procedure. Genes with internal restriction sites for any of these two enzymes were subcloned into the pUA1108 vector using the HiFi DNA assembly kit (New England Biolabs), following the manufacturer's protocol. Cloned genes were overexpressed in *E. coli* BL21-CodonPlus(DE3)-RIL (Stratagene) cells and the resulting His-tagged proteins were purified following the previously described protocol for LexA proteins (44). EMSAs were carried out using 100 bp-long DNA probes, which were generated using two complementary synthetic oligos centered on predicted binding-motifs and performing PCR with M13 universal digoxigenin-labeled primers (Supplementary Table S1) (9). EMSAs were performed on a mixture containing 25 ng of each digoxigenin-marked DNA probes and 30 nM of the purified Bacteroidetes SOS regulators as described previously (9). For competition assays, 400-fold molar excess of the same unlabeled probe was used as a specific competitor fragment (9). Samples were loaded onto 6% non-denaturing Tris-glycine polyacrylamide gels and DNA-protein complexes were detected using the manufacturer's protocol (Roche NimbleGen) (9). The DNA sequencing of all genes and probes was carried out by Macrogen.

### RT-qPCR gene expression analyses

Gene expression was determined by RT-qPCR as previously described (45), using Lightcycler RNA Master SYBR green I (Roche) on a Lightcycler 480 instrument (LC480; Roche), following the manufacturer's instructions. Exponential cultures of *S. agarivorans* DSM 23515 and *P. actiniarum* DSM 19842 were grown in Marine Broth at 28°C, and cultures of *E. anophelis* DSM 29660 were grown in Tryptone Soya Broth at 28°C. Cultures were inoculated with 7.5 μg/ml mitomycin C (Sigma-Aldrich), a concentration that has been frequently used in the past to study the SOS response in multiple organisms (3,46–51). After 3 h, RNA samples were extracted using the RNeasy mini-kit (Qiagen) following the manufacturer's instructions. Specific oligonucleotides were used to validate the expression of genes of interest (Supplementary Table S1). The relative mRNA concentrations of selected genes were determined according to a standard curve generated by amplifying a fragment of the *gyrB* gene. This gene is one of the most stable internal controls for RT-qPCR in multiple organisms, and it is known not to be inducible by DNA damage in *E. coli* (52–56). The gene expression factor was calculated as the ratio of the normalized mRNA concentration of each target gene in the treated strain versus the non-treated strain.

## RESULTS

### Two divergent DNA motifs define SOS-like regulatory networks in the Bacteroidetes

The presence of LexA homologs in the Bacteroidetes had been cursorily assessed when few complete genomes for this phylum were available (1). To ascertain whether members of this phylum encode LexA homologs, we used a reference dataset of experimentally-validated LexA proteins (Supplementary Table S2) to query the proteins encoded by all the Bacteroidetes complete genomes available in NCBI RefSeq with BLASTP. No significant hits were obtained, confirming the previous observation that Bacteroidetes genomes do not encode LexA homologs. However, BLAST searches using the *E. coli* UmuDC and DinB proteins revealed the presence of putative translesion synthesis polymerases in several members of this phylum (Supplementary Table S3). Given their high mutagenic activity, these polymerases tend to be tightly regulated (15).

**Figure 1.**
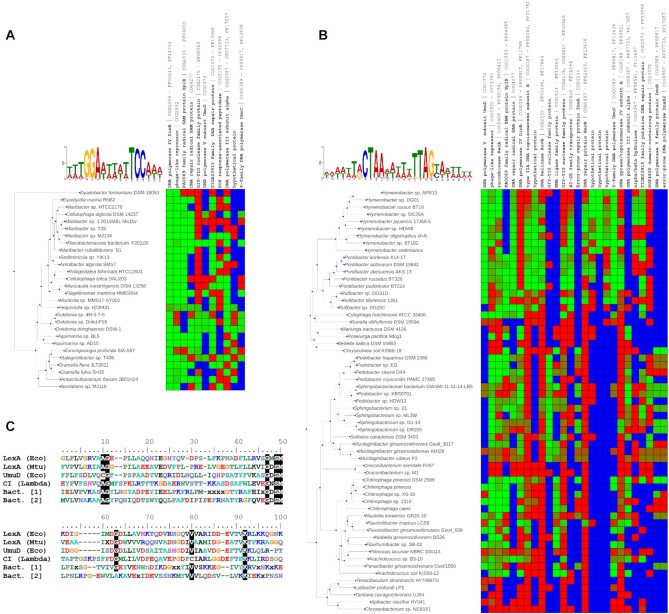
Comparative analysis of SOS Bacteroidetes regulons. (**A**) Sequence logo of the GGA-N5-TTC motif identified by MEME upstream of genes encoding UmuC/DinB proteins. CGB-generated heatmap of the posterior probability regulation for orthologous groups using the identified motif. Cells are colored from green (regulation) to red (no regulation), with blue indicating absence of ortholog. Only genes predicted to be regulated in at least 15% of the species analyzed are shown. (**B**) Sequence logo of the CTAA-N5-TTAG motif identified by MEME upstream of genes encoding UmuC/DinB and UmuD proteins. CGB-generated heatmap of regulation for orthologous groups using the CTAA-N5-TTAG motif. Color scheme is the same as in panel A. Only genes predicted to be regulated in at least 15% of the species analyzed are shown. (**C**) Comparative analysis of S24 peptidase domains. Multiple sequence alignment including the C-terminal segment of the *E. coli* (Eco) LexA and UmuD sequences, the *Mycobacterium tuberculosis* (Mtu) LexA sequence, the Lambda phage CI repressor sequence and the consensus sequences of the two Bacteroidetes SOS regulator clusters.

To investigate their possible regulation in the Bacteroidetes, we implemented a computational pipeline to detect orthologs of these proteins, automatically infer putative binding motifs and assess the regulatory network such motifs control (Supplementary Figure S1). We used HMMER to search Bacteroidetes proteomes using the HMM profiles for the COGs mapping to *E. coli* UmuD (COG1974) and DinB/UmuC (COG0389) (Supplementary Table S4). We obtained the nucleotide sequence upstream of the genes encoding putative UmuD and DinB/UmuC homologs and used MEME to detect overrepresented motifs in these sequences (Supplementary Data S3, Supplementary Data S4). MEME returned two significant motifs with apparent palindromic structure (Supplementary Figure S2), and these were subsequently refined by activating the palindromic constraint in MEME (Figure 1A, B). One of the motifs (GGA-N5-TCC) was identified only in the upstream regions of DinB/UmuC-homolog encoding genes, whereas the other motif (CTAA-N5-TTAG) was detected in the upstream regions of both DinB/UmuC- and UmuD-homolog encoding genes.

The detection, across multiple species, of well-defined motifs in the upstream region of genes encoding homologs of different core SOS proteins strongly suggested that these motifs could define a SOS regulatory network. To investigate this possibility we used both motifs to perform comparative genomic analyses of their putative regulatory network in the Bacteroidetes species in which the motifs had been identified. The results of the comparative analysis with the GGA-N5-TCC motif (Figure 1A) revealed a putative regulatory network dominated by genes encoding DinB homologs (COG0389), often associated with the gene encoding the DNA polymerase III alpha subunit (COG0587) in an operon arrangement that has been reported before in multiple SOS systems (40).

The conserved elements of this putative network also encompass the SplB radical SAM protein involved in DNA repair (COG1533), which has been shown to be SOS regulated in several species (57,7,10), as well as a two-gene operon encoding a putative DNA base excision repair system (COG1573-COG4277) involving a uracil-DNA glycosylase (UDG) (7). Other putative members of the inferred regulatory network include a *umuDC* operon (COG0389-COG1974) (40) and a SOS-response associated peptidase (COG2135) regulated by LexA in the Balneolaeota (10,58). Evidence of regulation for other genes encoding canonical SOS proteins (3), such as RecA (COG0468) and the mismatch repair enzymes UvrC (excinuclease; COG0322) and UvrD (helicase; COG0210) is also found in several species (Supplementary Figure S3).

The comparative analysis with the CTAA-N5-TTAG motif outlined a canonical SOS regulatory network, with core SOS genes (*umuDC*, *recA*, *recN*, *dinB*, *ruvB* and *splB*) presenting conserved instances of this motif in their upstream region. Genes encoding additional SOS proteins also showed substantial evidence of regulation by this motif in several Bacteroidetes species. These include the mutagenic cassette *imuA-imuB-dnaE2* (COG4544-COG0389-COG0587) (40) and the UDG DNA base excision repair system enzymes (COG1573-COG4277). The analysis also reveals evidence of regulation in several species of this phylum for the genes encoding the aforementioned SOS-associated peptidase (COG2135), the single-stranded binding protein Ssb (COG0629), the RecG DNA translocase (COG1200), the RecQ helicase (COG0514) and the DNA repair protein RadC (COG2003) (59) (Supplementary Figure S4).

In addition, the analyses with both motifs identified other proteins that could be potentially associated with a response to DNA damage (Figure 1AB). In particular, evidence of regulation under both motifs was observed for an GIY-YIG domain-containing exonuclease (COG0847) with homology to the proofreading subunit of DNA polymerase III, and for a phage-like repressor (COG2932). Furthermore, putative regulation by the CTAA-N5-TTAG motif was also consistently detected for an operon encoding DNA topoisomerase IV subunits A (COG0188) and B (COG0187). For both motifs, two or more motif instances were identified in the promoter region of several putatively regulated genes, a common feature in the SOS regulatory network (Supplementary Table S5) (3,7,60). Overall, these results are consistent with, and strongly suggestive of, SOS regulatory networks mediated via the identified motifs in the Bacteroidetes.

### A novel family of LexA-like repressors in the Bacteroidetes

The detection of putative SOS regulatory networks in the Bacteroidetes implicitly entailed the presence of transcriptional regulators targeting the identified motifs. Given the absence of canonical LexA homologs, an obvious candidate for this role were UmuDAb-type proteins, which have been described to target palindromic motifs (21), but no DNA-binding domains were detected in any of the Bacteroidetes UmuD homologs identified in the comparative analysis. Furthermore, several species with motifs identified upstream of *dinB* lack UmuD homologs (Supplementary Table S6), suggesting that a UmuD-like protein is not the regulator targeting these *dinB* promoters.

As mentioned above, a salient feature of the comparative analyses in Figure 1AB was the presence of an ortholog group mapping to COG2932 (Phage repressor protein C). Members of this ortholog group were present in all the species in which the GGA-N5-TCC and CTAA-N5-TTAG motifs were identified, and the genes coding for them presented strong evidence of regulation under both motifs (Figure 1A, B). This suggested that proteins from this orthologous group might be regulating the putative SOS regulatory networks defined by these two motifs. BLASTP searches against representative genomes from all other orders in the Bacteria domain did not return any significant matches, indicating that these putative transcriptional regulators are exclusive to the Bacteroidetes phylum.

A feature common to all SOS-like S24-family regulators (LexA, HdiR and UmuDAb) is their ability to undergo self-catalytic cleavage mediated by RecA nucleoprotein filaments. The sequence determinants for this process are well-conserved in the S24 serine peptidase family and have been amply documented (61). They encompass the presence of an Ala-Gly (Cys-Gly in UmuD) peptide bond that defines the cleavage site and a structurally adjacent Ser-Lys catalytic dyad (61). To investigate whether these sequence features were conserved in the putative SOS regulators mapping to COG2932 (Figure 1AB), we performed a multiple sequence alignment including the C-terminal regions of these proteins and of S24 family proteins for which the structure of the C-terminal domain has been experimentally determined.

The alignment (Figure 1C; Supplementary Data S5) revealed the presence of two distinct groups of sequences among these putative SOS regulators, and highlighted that all the key residues involved in the autocatalytic cleavage of S24 peptidases are conserved in both groups. To perform their regulatory function, S24-family regulators use different variants of the HTH DNA-binding domain (62). HHpred searches using a multiple sequence alignment of the putative Bacteroidetes SOS regulators N-terminal region matched members of the penta-helical HTH motif characteristic of phage repressors (PF01381), and subsequent search with HMMER identified this domain in all these putative SOS regulators (Supplementary Table S7). The data hence indicate that these putative Bacteroidetes SOS regulators encompass an S24 peptidase domain, containing the conserved residues involved in RecA-mediated self-catalytic cleavage of S24 peptidases. This supports the hypothesis that these proteins contain functional S24 peptidase domains, capable of undergoing self-catalytic cleavage mediated by RecA nucleoprotein filaments. Furthermore, the presence of an N-terminal HTH DNA-binding domain indicated that, like other S24 family members, these proteins could potentially bind DNA and operate as transcriptional regulators.

### Phylogeny and structure of putative Bacteroidetes SOS regulators

**Figure 2.**
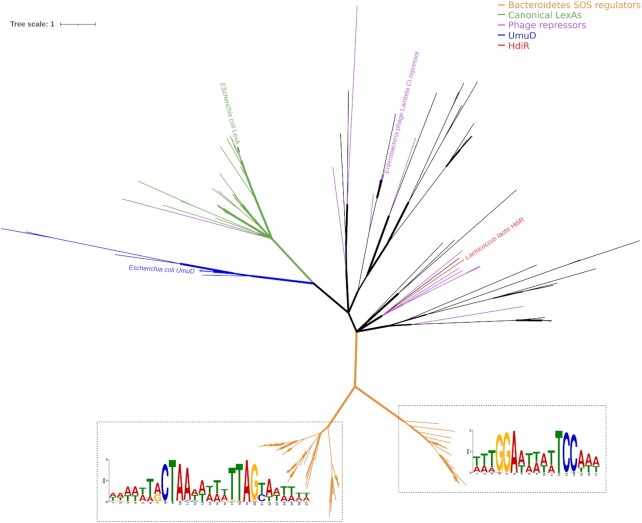
Unrooted Bayesian consensus tree of S24 peptidase family protein sequences. Sequences of putative SOS regulators (orange) were combined with the representative sequences for COG1974 (green for LexA and blue for UmuD) and COG2932 available in the COG database. Phage repressor coloring (purple) was assigned to COG2932 representatives if close homologs could be detected (>95% sequence similarity) in complete bacteriophage genomes. Branch width denotes support values. The inferred binding motifs for the Bacteroidetes SOS regulators are superimposed on the corresponding clades in the tree.

To elucidate the relationship between these putative Bacteroidetes SOS regulators and other S24 family members known to regulate SOS-like networks, we inferred their phylogeny using a multiple sequence alignment of these proteins and representative members of COG1974 (encompassing *E. coli* LexA and UmuD) and COG2932 (encompassing *L. lactis* HdiR and the repressors of *Enterobacteria* phage Lambda and *Salmonella* phage P22). The resulting tree (Figure 2; Supplementary Data S6) shows that the putative Bacteroidetes SOS regulators define a well-supported clade, clearly differentiated from those defined by COG1974 and COG2932 representatives. This result is in contrast with HdiR, which forms a well-defined cluster with *Streptococcus* phage repressors. The tree also reveals that these putative Bacteroidetes SOS regulators form two distinct clusters with significant intercluster distance, indicative of an ancient evolutionary split. Furthermore, the promoter regions of the genes encoding members of these two clusters presented predicted binding sites for the GGA-N5-TCC or CTAA-N5-TTAG motifs (Supplementary Table S8) in the comparative analyses of Figure 1AB, suggesting that the evolution of these DNA motifs was associated to the diversification of the putative Bacteroidetes SOS regulators in these two clusters.

Taken together, these data suggested that the putative Bacteroidetes SOS regulators define a novel family of LexA-like proteins controlling a conventional SOS network in multiple Bacteroidetes genera. Furthermore, even though they share a common ancestor, these LexA-like regulators show evidence of ancient diversification, leading to their targeting of distinct palindromic motifs in the Bacteroidetes.

**Figure 3.**
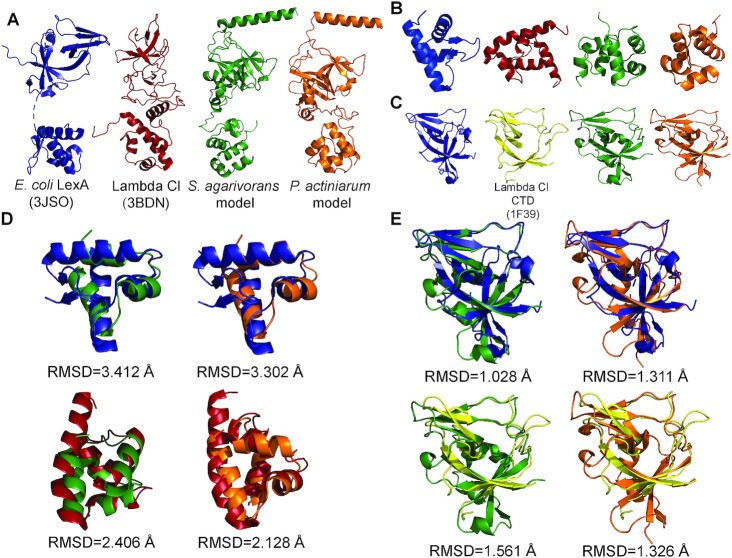
Comparisons of experimentally determined and calculated models of S24-family repressors. (**A**) X-ray crystal structures of full-length *E. coli* LexA (blue; PDB ID: 3JSO) and Lambda CI (red; PDB ID: 3BDN) compared to Robetta-calculated models of Bacteroidetes SOS repressors from *S. agarivorans* (green) and *P. actiniarum* (orange). (**B**) N-terminal domains (NTDs) of the proteins in panel A color-coded identically. (**C**) C-terminal Domains (CTDs) of the proteins in panel A, color-coded identically with the exception of the Lambda Cl CTD (yellow; PDB ID: 1F39), which is poorly resolved in the full-length structure (PDB ID: 3BDN). (**D**) Superpositions of NTDs and their RMSD values color-coded as in panel A. The numbers of superposed Cαs are as follows: 33 Cαs for *E. coli* and *S. agarivorans*; 38 Cαs for *E. coli* and *P. actiniarum*; 55 Cαs for Lambda Cl and *S. agarivorans*; and 33 Cαs for Lambda Cl and *P. actiniarum*. (**E**) Superpositions of CTDs and their RMSD values color-coded as in panel B. The number of superposed Cαs are as follows: 63 Cαs for *E. coli* and *S. agarivorans*; 67 Cαs for *E. coli* and *P. actiniarum*; 61 Cαs for Lambda Cl and *S. agarivorans*; and 56 Cαs for Lambda Cl and *P. actiniarum*.

To further explore the relationship between putative Bacteroidetes SOS regulators and other S24 family members, we performed structural predictions of two representative species from each cluster (*S. agarivorans* DSM 23515 [WP_075324850.1] and *P. actiniarum* DSM 19842 [WP_025606010.1]) using the Robetta suite (Figure 3; Supplementary Data S7). The predicted three-dimensional fold of each protein is composed of three domains: an N-terminal domain, an unstructured linker region, and a C-terminal domain, similar to the observed three-dimensional folds of both *Enterobacteria* phage Lambda CI (full length PDB ID: 3BDN; C-terminal domain only PDB ID: 1F39) and *E. coli* LexA (PDB ID: 3JSO) (Figure 3A). We then compared the predicted C- and N-terminal domains of the representative species to the crystallized C- and N-terminal domains of *Enterobacteria* phage Lambda CI and *E. coli* LexA (Figure 3BCDE).

Qualitatively, and consistent with HHpred results, the folds of the structurally-predicted C-terminal domains (CTDs) bore a strong resemblance to the folds of both *Enterobacteria* phage Lambda CI and *E. coli* LexA CTDs, with the exception of a terminating α-helical segment of unknown function in the putative Bacteroidetes SOS regulators (Figure 3CE). In contrast, the structurally-predicted N-terminal domains (NTDs) of the putative Bacteroidetes SOS regulators were dissimilar from the *E. coli* LexA NTD (Figure 3C, D). In fact, these calculations predict that the NTDs of the *S. agarivorans* and *P. actiniarum* SOS regulators are multi-helical HTH domains (composed of only α-helices) strongly resembling the *Enterobacteria* phage Lambda CI NTD rather than the winged-helix HTH NTD (composed of both α-helical and β-sheet segments) that is characteristic of *E. coli* LexA.

These observations are underscored by RMSD calculations of the N-terminal superpositions of the known and predicted NTD structures (Figure 3D), in which only 3 α-helices of calculated NTDs superpose modestly with a strong mismatch of the *E. coli* LexA winged-helix NTD, compared to a satisfactory superposition of all 4 α-helices of the calculated NTDs with the Lambda CI NTD. In contrast, RMSD calculations of the CTD superpositions (Figure 3E) are excellent irrespective of the organism of origin. These results remained qualitatively the same regardless of whether the three-dimensional structural predictions were calculated based on homology or through *ab initio* strategies. Thus, the three-dimensional fold of the N-terminal DNA-binding domain of the Bacteroidetes SOS regulators is predicted to have striking similarity to the N-terminal multi-helical HTH domain of *Enterobacteria* phage Lambda CI.

### Sequence-specific binding and regulation of SOS genes by Bacteroidetes SOS regulators

Bioinformatics analyses indicated that a new family of putative Bacteroidetes SOS regulators controlled conventional SOS regulons using two distinct motifs. These analyses originated with homology searches of error-prone polymerases DinB and UmuD, leaving open the possibility that homologs of these regulators in Bacteroidetes species not harboring these error-prone polymerases might target other motifs. To ascertain whether this was the case, we identified additional homologs of these regulators in the Bacteroidetes using BLASTP (Supplementary Table S9) and we performed motif discovery with MEME using their promoter regions. As expected, MEME elicited only the two previously identified DNA motifs, which associate preferentially with members of the two putative Bacteroidetes SOS regulators clusters, as illustrated in Figure 2 (Supplementary Figure S5).

**Figure 4.**
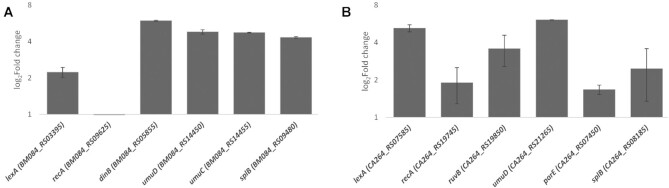
*In vivo* validation of the SOS Bacteroidetes regulators. Induction factor (log_2_) of (**A**) *S. agarivorans* and (**B**) *P. actiniarum* genes as the ratio of normalized mRNA between cultures treated with mitomycin C and untreated cultures, determined by RT-qPCR. The error bars represent the standard error of the mean of two independent biological replicates.

To assess whether the predicted regulation of genes by these putative SOS regulators had a SOS-like regulatory effect, we analyzed the expression of several genes predicted to be regulated in each species under SOS-inducing conditions by RT-qPCR. The results of the RT-qPCR assays following mitomycin C treatment for both species (Figure 4) show significant induction for the genes encoding the *P. actiniarum* and *S. agarivorans* putative SOS regulators, as well as for canonical and putative SOS genes predicted to be regulated in the comparative analysis (Figure 1AB). The only exception is the *S. agarivorans recA* gene (Figure 4A), which does not show apparent induction as reported before for other organisms (48,49,63). In both species, the largest induction values correspond to genes encoding type IV and type V error-prone polymerases.

**Figure 5.**
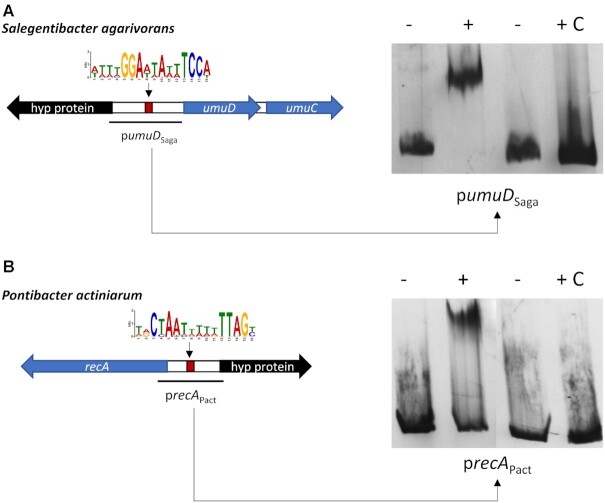
Binding specificity of SOS Bacteroidetes regulators. EMSA with purified (**A**) *S. agarivorans* (*S_aga_*) and (**B**) *P. actiniarum* (*P_act_*) putative SOS regulator proteins on the promoter region of the genes encoding UmuD and RecA orthologs, respectively. The ‘–’ symbol denotes absence of protein and ‘+’ the presence of protein in the mixture. The ‘C’ symbol indicates the presence of unlabeled competitor DNA.

To determine whether the putative SOS regulators in each of the two clusters bound specifically to their predicted motifs, we purified the corresponding proteins for two representative species from each cluster (*S. agarivorans* DSM 23515 [WP_075324850.1] and *P. actiniarum* DSM 19842 [WP_025606010.1]) and performed electromobility-shift assays (EMSA) on promoters (*umuD* and *recA*, respectively) predicted to be regulated by each protein using unlabeled promoter DNA as a control. EMSAs with both putative SOS regulators (Figure 5) reveal a retardation band consistent with binding of the protein to the target promoters containing predicted binding sites. Furthermore, the retardation band is abolished by the addition of unlabeled promoter DNA, demonstrating that both regulators bind specifically to these promoter sequences.

**Figure 6.**
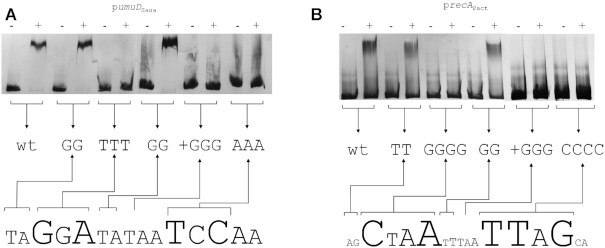
*In vitro* validation of the SOS Bacteroidetes regulators binding motifs. EMSA with purified (**A**) *S. agarivorans* (*S_aga_*) and (**B**) *P. actiniarum* (*P_act_*) putative SOS regulator proteins on the wild-type and site-directed mutagenesis variants of the promoter region of the genes encoding UmuD and RecA orthologs, respectively. The ‘–’ symbol denotes absence of protein and ‘+’ the presence of protein in the mixture.

To further delineate the region specifically bound by these putative SOS regulators and identify the specific regions involved in binding, we performed EMSAs coupled with site-directed mutagenesis on both promoters. The results demonstrate that the *S. agarivorans* putative SOS regulator (WP_075324850.1) binds specifically to the palindromic GGA-N5-TCC motif (Figure 6A). Substitutions on either motif dyad, as well as changes in spacer length, of the motif completely abolished binding, whereas substitutions in spacer and dyad-adjacent positions had no significant effect on binding. Similarly, the *P. actiniarum* putative SOS regulator (WP_025606010.1) was found to bind specifically to the CTAA-N5-TTAG motif (Figure 6B). As in the case of *S. agarivorans*, substitutions on either motif dyad and changes to the spacer resulted in the abolishment of binding by the putative regulator, while substitutions in dyad-adjacent and spacer positions had no effect. Furthermore, electromobility-shift assays on the upstream region of the genes encoding the *P. actiniarum* and *S. agarivorans* putative SOS regulators, containing instances, respectively, of the GGA-N5-TCC and CTAA-N5-TTAG motifs, determined that both proteins are capable of binding their respective promoters (Supplementary Figure S6).

Together, these results demonstrate that the two putative Bacteroidetes SOS-like regulators assessed here control the expression of canonical and putative SOS genes in response to DNA-damage by binding specifically to their predicted binding motifs and that, like LexA, they act as global transcriptional repressors. The presence of homologs for these two proteins and of predicted SOS-like networks using their respective motifs in multiple Bacteroidetes species suggests that these proteins are likely bona fide regulators of the SOS response in the Bacteroidetes phylum.

### Distribution of the SOS response and its regulators in the Bacteroidetes

The results presented above established the presence of two clearly differentiated members of a novel family of regulators controlling large SOS regulatory networks via distinct motifs. Homology searches, however, revealed that several Bacteroidetes clades did not harbor homologs of these regulators. To investigate the evolutionary history of this transcriptional response in the Bacteroidetes, we performed Bayesian phylogenetic inference on RecA protein sequences from representative species harboring these novel SOS regulators and representative members of all Bacteroidetes orders. On the inferred tree, we annotated the presence of SOS regulators mapping to the GGA-N5-TCC and CTAA-N5-TTAG motif clusters, their putative regulation by the corresponding motif and the inferred size of the regulon based on the number of known SOS genes with evidence of regulation under the corresponding motif.

**Figure 7.**
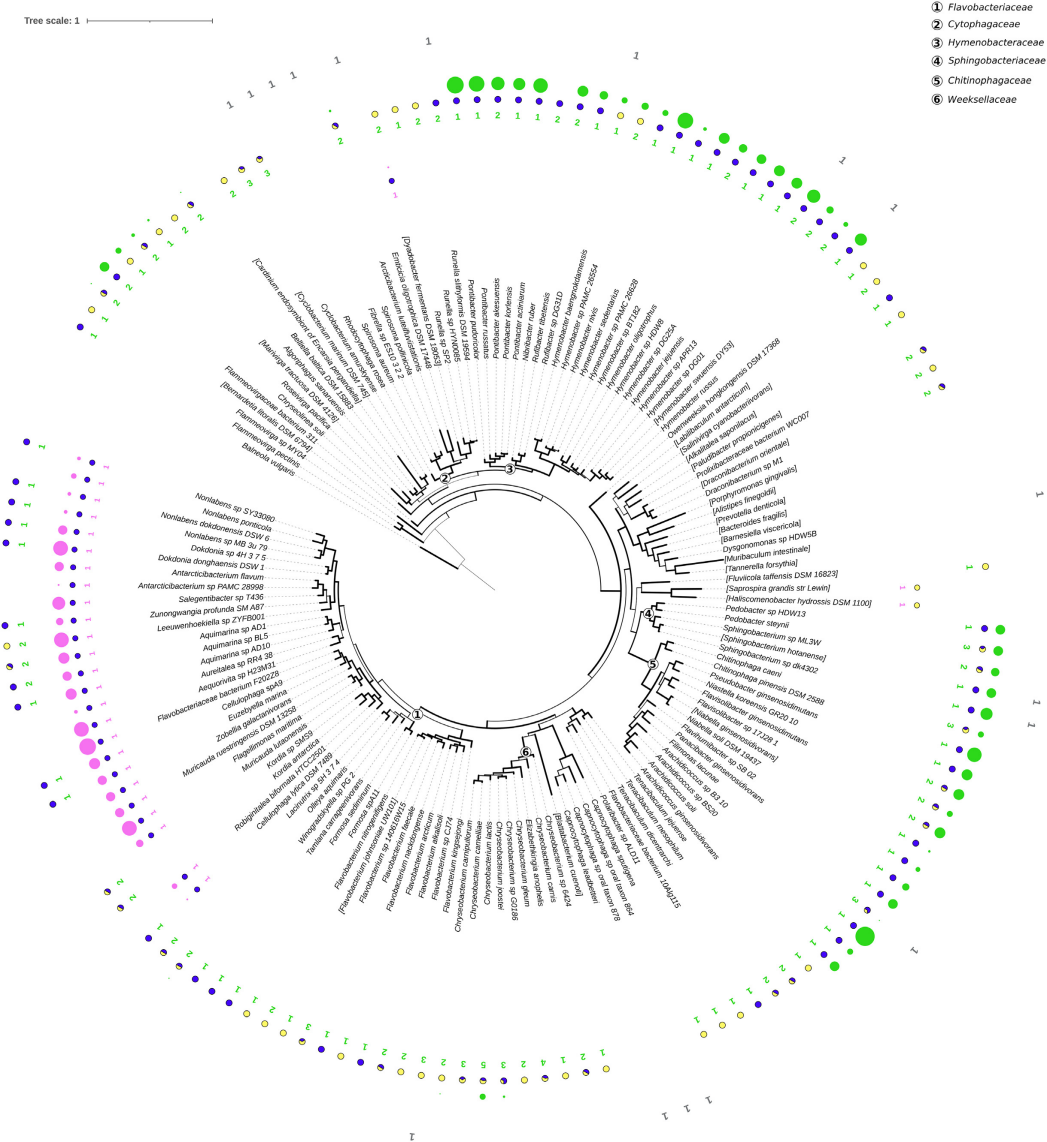
Rooted Bayesian consensus tree of Bacteroidetes RecA protein sequences. Branch width denotes support values. Next to each tip label, information for both Bacteroidetes SOS regulator types (pink/green for the regulators targeting GGA-N5-TTC and CTAA-N5-TTAG motifs, respectively) is represented with three items: colored numbers indicate the number of encoded SOS regulator genes; pie charts represent the fraction of regulated (blue) and not regulated (yellow) SOS regulators; colored circles denote the number of additional genes predicted to be regulated by the respective SOS regulators. Gray numbers stand for unclassified SOS regulators. Families of Bacteroidetes that are discussed in the text are highlighted with circled numbers. The tree was rooted using the *Balneolaeota vulgaris* RecA protein sequence as an outgroup.

The resulting tree (Figure 7, Supplementary Data S8), in broad agreement with previously reported Bacteroidetes phylogenies (64), revealed remarkable diversity in the distribution of these two SOS regulator ortholog groups across the Bacteroidetes. Orthologs mapping to the CTAA-N5-TTAG group are widely distributed across the Bacteroidetes, whereas the GGA-N5-TCC group is found primarily in the *Flavobacteriaceae* family. Both groups of orthologs are absent in the Bacteroidales order, and in the *Capnocytophaga* and *Cytophagaceae* sequence divergence in the identified homologs prevented their unequivocal assignment to either cluster. Regulon size varies significantly, but is fairly cohesive within phylogenetic clades, with several families presenting large (8.90 ± 3.27 operons) regulons under the CTAA-N5-TTAG (*Hymenobacteraceae*, *Sphingobacteriaceae* and *Chitinophagaceae*) and GGA-N5-TCC (*Flavobacteriaceae*) motifs. Interestingly, regulon size is markedly small in species related to clades where SOS regulators are absent or highly diverged, suggesting that loss of regulation is associated with regulon contraction. Within the *Flavobacteriaceae*, a significant number of species present homologs mapping to both ortholog groups, but the predicted SOS regulon is under control of the GGA-N5-TCC group regulator.

**Figure 8.**
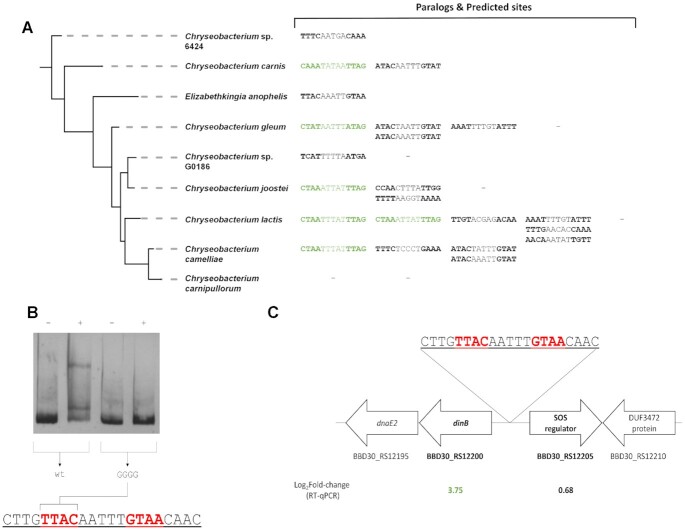
Analysis of divergent palindromes in the promoter region of apparently unregulated Bacteroidetes SOS regulators. (**A**) Inferred phylogeny for the *Weeksellaceae* family (Figure 7) showing predicted binding sites (MEME predicted CTAA-N5-TTAG sites and identified perfect palindromes) for each SOS regulator paralog. MEME predicted sites are highlighted in green. (**B**) Electromobility-shift assays with the purified *E. anophelis* SOS regulator on the wild-type and site-directed mutagenesis variants of the promoter region of its encoding gene. The ‘–’ symbol denotes absence of protein and ‘+’ the presence of protein in the mixture. (**C**) Gene expression factor (log_2_) of *E. anophelis* genes after mitomycin C induction as determined by RT-qPCR. The genomic environment of the SOS regulator encoding gene is also shown.

The analysis also revealed extensive paralogy in the CTAA-N5-TTAG ortholog group, with some species in the *Weeksellaceae* family harboring up to five homologs of these SOS regulators. Paralogy in the CTAA-N5-TTAG group regulators is positively correlated (Spearman ρ = 0.35, *P* < 0.001) with the loss of the CTAA-N5-TTAG motif associated with this ortholog group. This suggested that duplication events might be facilitating divergence in SOS regulator homologs and their associated binding motif. To investigate this hypothesis, we examined the promoter region of Bacteroidetes SOS regulators that did not contain instances of the CTAA-N5-TTAG motif for the presence of alternative palindromes that could constitute divergent binding motifs. We identified several instances of putative diverged binding motifs following the dyad-spacer palindromic structure of the CTAA-N5-TTAG motif (Figure 8A; Supplementary Table S10). To assess whether these predicted palindromes could be functional binding sites for their cognate SOS regulators, we experimentally confirmed the specific binding of the *E. anophelis* strain DSM 29660 SOS regulator (WP_078407279.1) to the predicted binding site TTAC-AAATT-GTAA in its promoter sequence (Figure 8B). We also determined that the gene encoding this transcriptional regulator and its tandem opposite, encoding an error-prone polymerase, are induced by mitomycin C treatment (Figure 8C).

## DISCUSSION

### A canonical SOS response under control of non-canonical LexA proteins

Comparative analyses have established that error-prone polymerases constitute the conserved core SOS response across bacteria, presumably due to the need to mitigate the high mutagenic load of unregulated error-prone polymerases (1,10,15). Leveraging this insight, we performed a systematic comparative analysis of error-prone polymerase regulation in the Bacteroidetes. Motif discovery (Figure 1AB) elicited two putative regulatory motifs (GGA-N5-TTC and CTAA-N5-TTAG) upstream of genes encoding error-prone polymerases in the Bacteroidetes. Comparative genomics analyses of the putative regulatory networks encoded by these motifs (Figure 1AB) revealed a large regulatory network encompassing many well-documented elements of the SOS response (*recA*, *dinB*, *umuDC*, *uvrC*, *uvrD*, *imuA-imuB-dnaE2*, *ssb*, *splB* and *radC*; (3,7,40,57,59)).

Beyond canonical SOS genes, the analysis also confirmed the regulation of several genes that have been previously identified as members of the SOS response only in a few species. The SOS regulation of a putative uracil-DNA glycosylase-based DNA base excision repair system has been reported in the Verrucomicrobia and the Alphaproteobacteria (7,65). Our findings in the Bacteroidetes suggest that uracil-DNA glycosylases may be a relatively common feature of the SOS response, aimed at addressing ribonucleotide misincorporation by SOS-induced error-prone polymerases. In a similar vein, regulation was also predicted for an operon encoding the A (*parC*) and B (*parE*) DNA topoisomerase IV subunits, and their induction by mitomycin C was confirmed by RT-qPCR (Figure 4A). The regulation of topoisomerases by the SOS response has been described in the *Vibrionaceae* (59,66), and the regulation of *parCE* in the Bacteroidetes indicates that topoisomerases may also be a recurring component of the SOS response, participating in the resolution of converging replication forks or acting as an alternative repair pathway for double-stranded breaks (67,68).

In the absence of bona fide LexA homologs, the comparative genomics analysis identified two orthologous groups of putative phage repressors, each one showing clear evidence of regulation by one of the identified motifs (Figure 1AB), as possible regulators of the inferred SOS-like networks. Both groups of repressors present the signature residues of S24 peptidases (Figure 1C), and we confirmed that exemplars of each group are DNA-damage inducible (Figure 4). Furthermore, both regulators are capable of binding specifically to their assigned motifs, and target genes were shown to be induced by mitomycin C treatment (Figure 4). Taken together, these results demonstrate that these S24-family phage-like repressors control the expression of SOS genes in multiple Bacteroidetes species. LexA proteins have been reported to act as transcriptional activators (69) and, in the absence of non-cleavable mutant data, it cannot be discarded that these S24-family phage-like repressors activate expression of SOS genes following DNA damage induction via a hitherto unknown mechanism. The sequence conservation of both S24 signature elements (the scissile Ala-Gly bond and the Ser-Lys catalytic dyad; Figure 1C) in these proteins and the robust overlay of their catalytic C-terminal domains with that of self-cleavable S24 enzymes (Figure 3E), however, strongly suggests that these S24-family phage-like repressors operate as SOS transcriptional repressors in this phylum.

DNA-damage inducible control of SOS genes by S24 peptidases other than LexA has been reported before in two bacterial families: the *Streptococcaceae* and the *Moraxellaceae*. However, in these families the corresponding S24-family regulators (HdiR and UmuDab) control only the induction of a minimal SOS regulon, composed primarily of error-prone polymerases (17,20). Their independent evolution in two unrelated bacterial clades suggests that regulation of error-prone polymerases by S24 peptidases is a convergent evolutionary process driven by the need to silence their expression in non-stressed conditions. Our results indicate that this convergent evolutionary process does not necessarily stop with the regulation of error-prone polymerases, but can continue with the incorporation of DNA repair and recombination enzymes into a full-fledged SOS response.

### Functional definition of SOS transcriptional repressors

The discovery of a group of repressors with structural similarity to phage repressors controlling a canonical SOS response in the Bacteroidetes poses relevant questions regarding the origin of this transcriptional response and its repressor. The origin of S24-family repressors previously shown to regulate SOS genes is well-defined. UmuDAb has been shown to cluster with regular UmuD proteins in the *Moraxellaceae* (70), whereas HdiR clearly clusters with *Streptococcus* phage repressors in Figure 2. In contrast, phylogenetic and multiple sequence alignment analyses of the Bacteroidetes SOS regulators and other S24 peptidases (Figure 1C, Figure 2) indicate that these regulators define a new family of S24 regulators restricted to the Bacteroidetes.

Structural comparisons with the reference S24 peptidase structures of *E. coli* LexA and *Enterobacteria* phage Lambda CI show marked divergence from key domains of the predicted structures of Bacteroidetes SOS regulators. The structurally-predicted C-terminal domains overlay remarkably well with both the LexA and CI C-terminal domains (Figure 3CE), responsible for dimerization and autocatalytic cleavage, suggesting that functional constraints have preserved the structure of this domain in spite of substantial sequence divergence, as has been noted before for LexA, UmuD and CI (71).

In contrast with the C-terminal domains, the structurally-predicted N-terminal domains of Bacteroidetes SOS regulators do not superpose well with the winged HTH N-terminal domain of LexA (Figure 3BD). Instead, these N-terminal domains contain four predicted α-helices that overlay well with the N-terminal domain of CI. This difference suggests that, like HdiR, these Bacteroidetes regulators are most likely of phage origin. In this context, it is worth noting that the C-terminal domain of these regulators harbors a predicted α-helix that is not present in LexA or CI structures. While CI repressors can target dyad motifs with variable spacing (72), LexA proteins are known to target motifs with rigid spacers, and this feature has been attributed to stabilizing contacts mediated by the wing of the winged HTH domain (12). Given the absence of such a wing in Bacteroidetes SOS regulators, but their consistent targeting of fixed-spacer motifs (Figure 6), we speculate that the additional C-terminal α-helix may be involved in dimer stabilization, leading to rigid recognition of fixed-spacer motifs.

**Figure 9.**
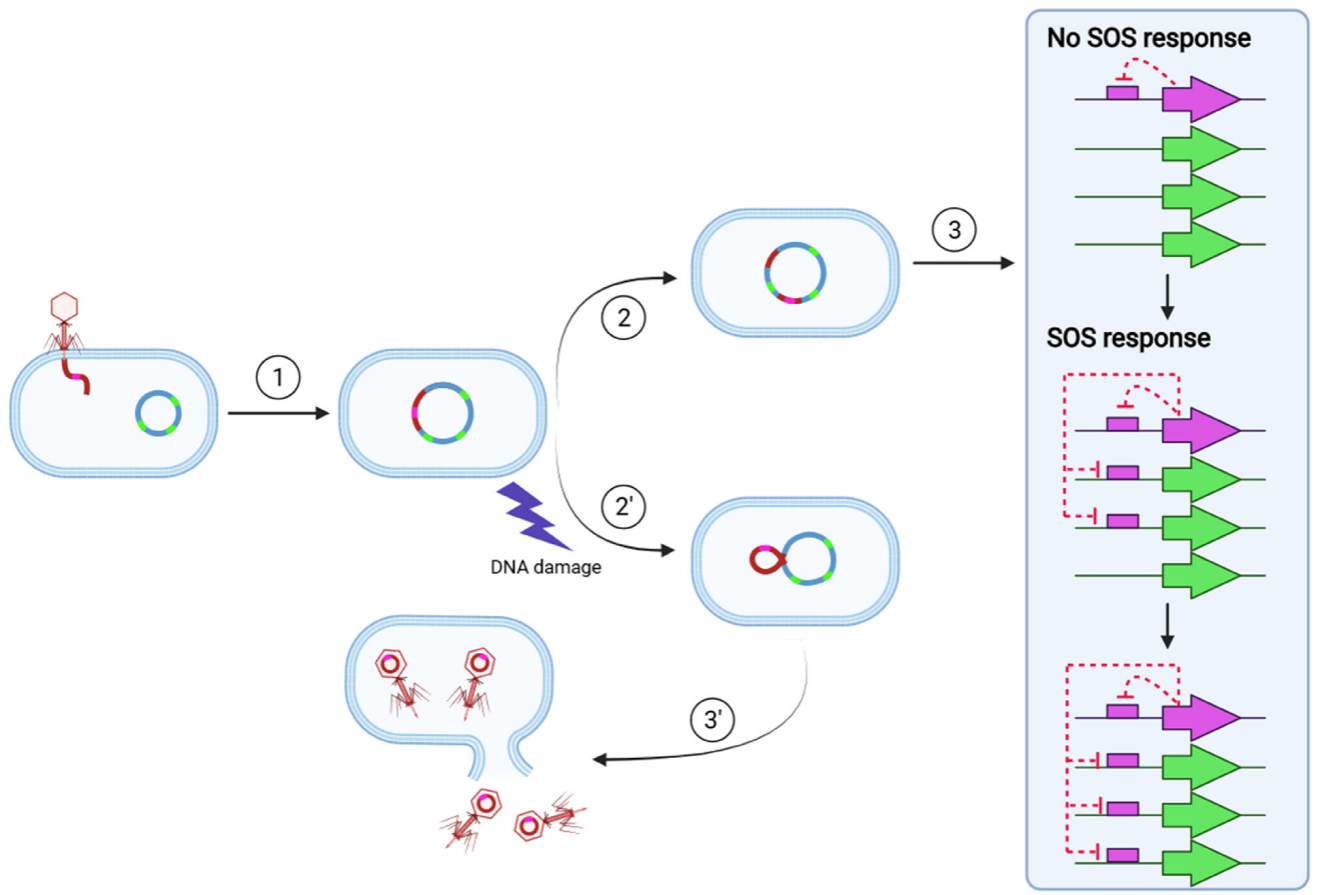
Uptake of the SOS regulatory network by a phage lytic cycle repressor. (1) A temperate phage infects a bacterial cell containing unregulated SOS genes (green) and integrates in its chromosome as a prophage. (2′ & 3′) Standard pathway for prophage lytic development under control of the phage repressor (pink). (2) Mutational events disrupt the prophage, rendering it inactive. (3) The prophage repressor (pink arrow) gradually takes up regulation of cellular SOS genes (green arrows), yielding a functional SOS response. This figure was constructed using BioRender templates.

Our results indicate that repressors of putative phage origin have taken up SOS regulatory functions at least twice (in the *Streptococcaceae* and the Bacteroidetes) through a process of convergent evolution. In the Bacteroidetes, this has resulted in phage-like repressors orchestrating a classical SOS response. Given that the canonical LexA is also a member of the S24 family, and that convergent evolutionary processes seem to be capable of replicating the conventional SOS response with alternative repressors, it is plausible to assume that the canonical LexA repressor may also have originated from the capture of a bacteriophage repressor by an early ancestor of extant bacterial groups (Figure 9).

Direct links between the SOS response and bacteriophages have been noted before (1). The lytic cycle of temperate phages is usually controlled by CI-like repressors, but in several instances the host LexA protein is used to repress lytic genes, either directly or through its interaction with repressors or anti-repressors (73–76). These examples, together with additional instances of LexA regulating the mobilization of other genetic elements (1), blur the lines regarding the definition of the SOS response. The common denominator in all these cases is the adaptive advantage of responding to DNA damage conferred by the LexA repressor both to bacterial cells (which can activate repair/bypass mechanisms) and to bacteriophages and other mobile genetic elements (which can seek out undamaged hosts). The here-reported ability of putative phage repressors to coordinate the regulation of DNA repair and translesion synthesis pathways indicates that the connection between the bacterial SOS response and bacteriophages is a two-way process, with both entities capable of co-opting the other's DNA damage response mechanisms.

In this context, and given the inability of structural information to clearly distinguish between the different transcriptional repressors involved, we advocate the adoption of a functional nomenclature. The term *lex* was first coined to designate the ‘locus for X-ray sensitivity’ corresponding to *lexA* in the *E. coli* genome (77) and, subsequently, the name *lexA* has been consistently used to refer to the DNA-damage inducible repressor of the SOS response. We hence propose to designate hereafter as ‘LexA’ those chromosomally-encoded repressors in control of a canonical SOS network (DNA repair, translesion synthesis and cell-cycle arrest), including the Bacteroidetes SOS repressors here described.

### Motif divergence and convergent evolution of SOS regulation

**Figure 10.**
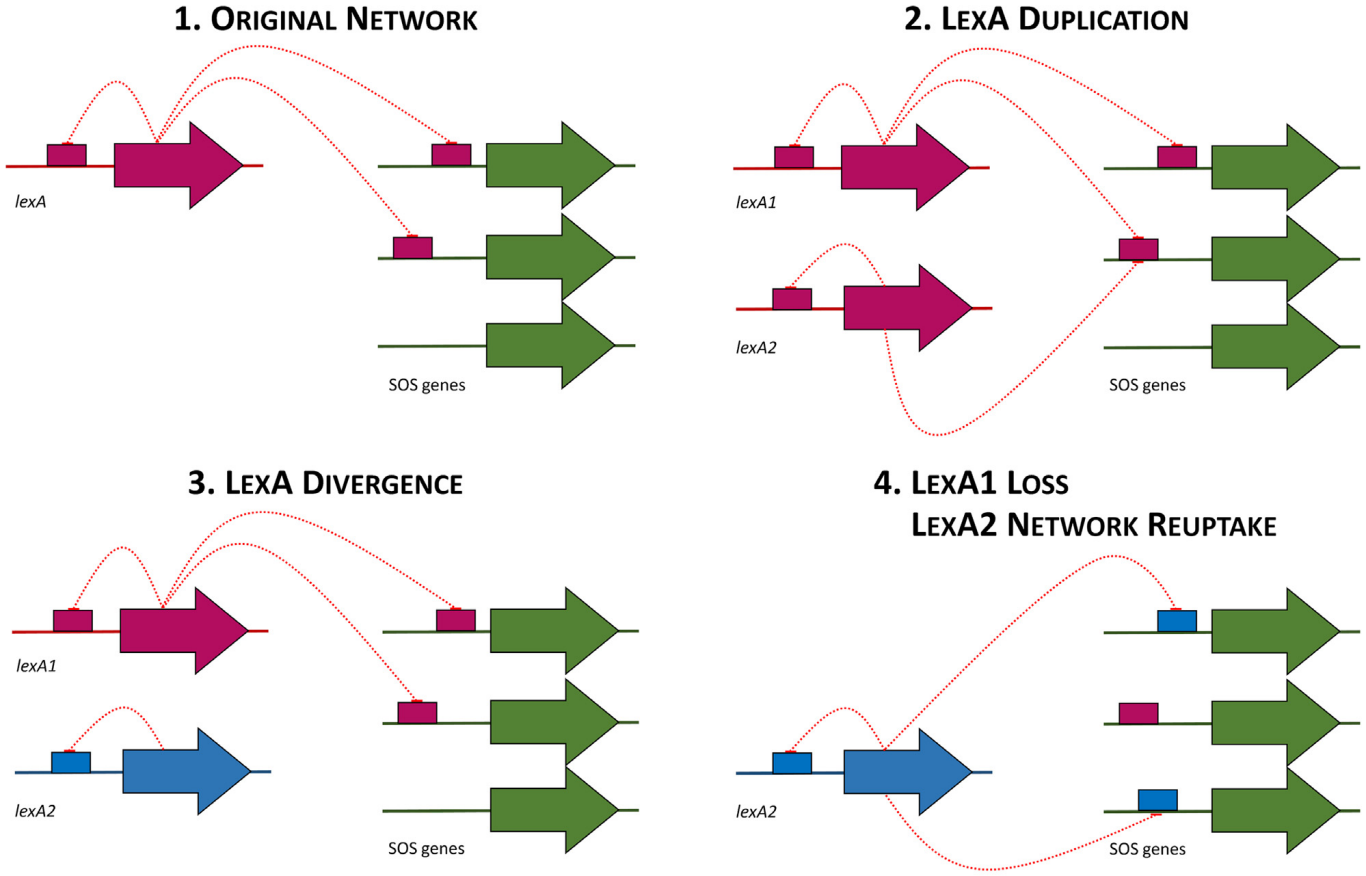
Model for SOS network evolution. (1) LexA controls the expression of the SOS network, regulating itself and other SOS genes. (2) A *lexA* gene duplication takes place. (3) One of the LexA proteins diverges, altering its LexA-binding motif. (4) Upon deletion of the primary LexA, the diverged LexA protein gradually reuptakes the regulon, using the novel LexA-binding motif.

A prominent feature of the SOS response is the documented tendency of the LexA transcriptional repressor to dramatically alter its binding motif throughout the course of evolution, with 17 distinct LexA-binding motifs reported to date in different bacterial groups (1,8,7,9,10). Hypervariability in the LexA-binding motif could in principle be explained by inherent lability in the LexA DNA-binding domain, but the LexA-binding motif has been documented to be extremely stable across broad phylogenetic groups, suggesting that other factors are involved (16,78,79). Recent and ancient duplications of the *lexA* gene have been reported in multiple bacterial groups, and shown to correlate with the emergence of novel motifs (63,75,80,8). Duplications in transcription factors can rapidly lead to motif divergence and the uptake of novel regulatory functions (81,82). In the case of LexA, it has been proposed that, after duplication, a diverged LexA protein may reuptake SOS regulatory functions following the loss of the primary *lexA* gene, resulting in an apparent change to the LexA-binding motif (7) (Figure 10).

The results reported here depict a strikingly similar picture for a novel SOS repressor that is not a homolog of LexA. As in the case of conventional LexA proteins, the Bacteroidetes LexA shows clear evidence of gene duplication, with several groups harboring multiple paralogs of the CTAA-N5-TTAG group LexA, as well as coexistence of Bacteroidetes LexA proteins targeting the GGA-N5-TCC and CTAA-N5-TTAG motifs in the same species (Figure 7). Furthermore, our data also demonstrate that degenerate variants of the CTAA-N5-TTAG motif are functional in clades with multiple parologs (Figure 8; Supplementary Table S10), and support the notion that gradual regulon reduction, and the eventual loss of regulation, are strongly associated with duplication events. The fact that the evolution of the SOS response in the Bacteroidetes, under control of a non-canonical LexA protein with a distinct DNA binding domain (Figure 3B, D), parallels that of previously reported SOS systems under control of canonical LexA proteins suggests that hypervariability in LexA-binding motifs is not due to specific DNA-binding particularities of the LexA repressor, but rather it is an intrinsic property of the SOS response.

## DATA AVAILABILITY

Python scripts used for obtaining and analyzing genomic data are available in the GitHub repository (https://github.com/ErillLab/SOS_Bacteroidetes/). All the genome and protein sequence data used in this work is available in the NCBI RefSeq database (https://www.ncbi.nlm.nih.gov/refseq/). The protein structures used in this study are available at the Protein Data Bank (https://www.rcsb.org/). The T-COFFEE multiple sequence alignment suite is available at http://www.tcoffee.org/. The HMMER suite is available at http://hmmer.org/. CGB is a software suite for comparative analysis of prokaryotic regulatory networks available in the GitHub repository (https://github.com/ErillLab/cgb/). The multiple sequence aligner CLUSTALW is available at http://www.clustal.org/. The PyMol molecular visualization suite is available at https://pymol.org/. The Robetta protein structure prediction service is available at https://robetta.bakerlab.org/. The phylogenetic inference software MrBayes is available at https://nbisweden.github.io/MrBayes/.

TrRosetta models for *S. agarivorans* DSM 23515 [WP_075324850.1] and *P. actiniarum* DSM 19842 [WP_025606010.1] are available in ModelArchive (https://www.modelarchive.org/) under accession numbers ma-gno0n and ma-l80i0, respectively.

Accession numbers for all other sequences and structures used in this study are provided in the supplementary materials.

## Supplementary Material

gkab773_Supplemental_Files
