# Supplementary material for: Non-canonical LexA proteins regulate the SOS response in the Bacteroidetes
Source: Nucleic Acids Res. 2021 Oct 6;49(19):11050–66. doi: 10.1093/nar/gkab773 (PMC8565304; doi:10.1093/nar/gkab773)
Supplement: gkab773_Supplemental_Files [file gkab773_supplemental_files.zip › Supplementary Figure 1.pdf]

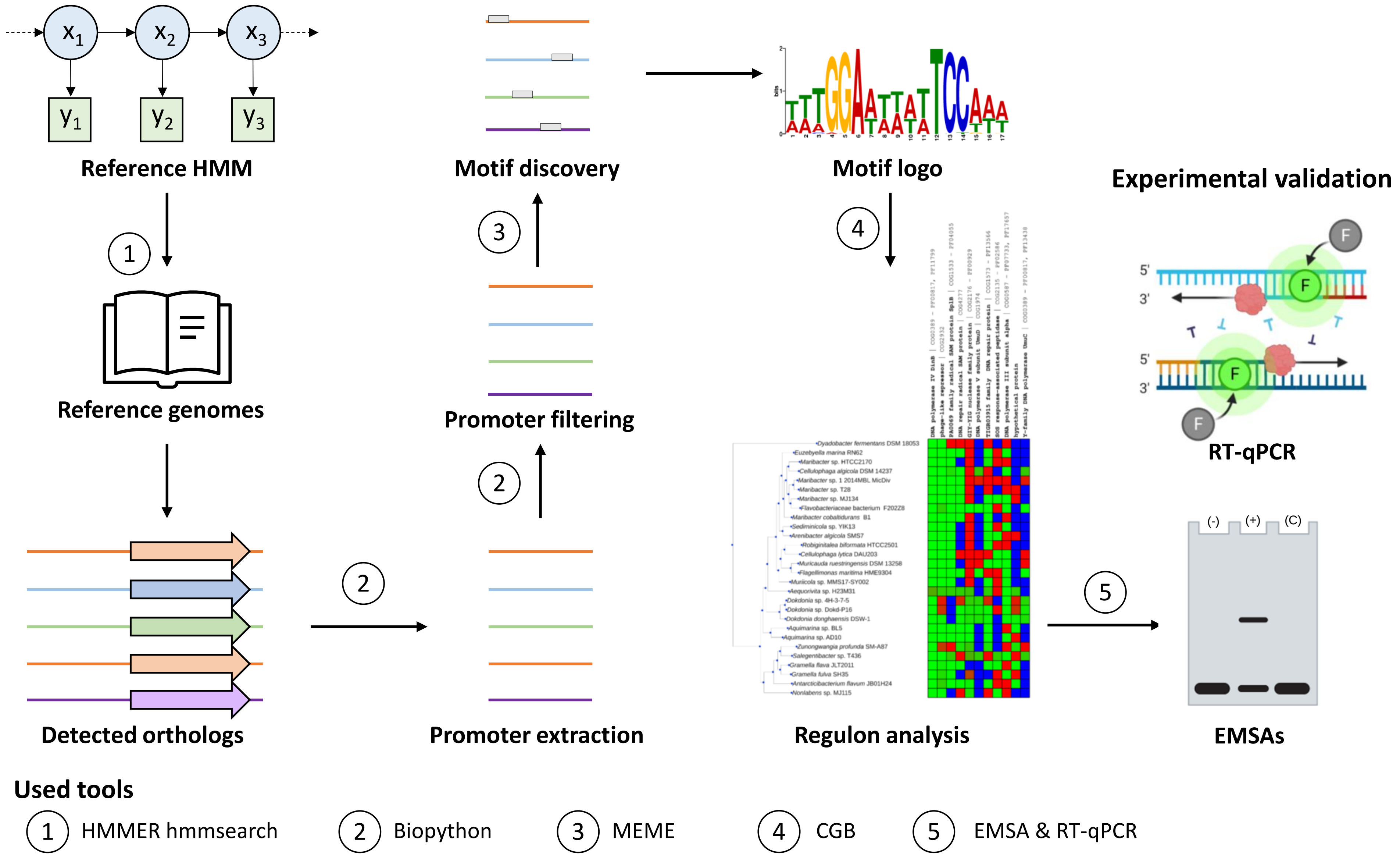

Figure S1: Flow diagram of the pipeline used in this work. (1) Hidden Markov Models for reference COGs are used to search reference genomes with hmmsearch in order to detect orthologs of genes likely to be regulated by the SOS response. (2) Biopython scripts are used to retrieve the corresponding promoter sequences and filter them by sequence similarity to generate a library of diverse promoters on which to perform motif discovery. (3) Motif discovery is performed on the promoter library using MEME. (4) Inferred motifs are used to elucidate putative regulatory networks using the CGB comparative genomics suite. (5) Binding of putative regulators and DNA damage induction are validated for target genes with identified sites for inferred motifs. This figure was constructed using some BioRender templates.
