# Supplementary material for: Non-canonical LexA proteins regulate the SOS response in the Bacteroidetes
Source: Nucleic Acids Res. 2021 Oct 6;49(19):11050–66. doi: 10.1093/nar/gkab773 (PMC8565304; doi:10.1093/nar/gkab773)
Supplement: gkab773_Supplemental_Files [file gkab773_supplemental_files.zip › Supplementary Figure 2.pdf]

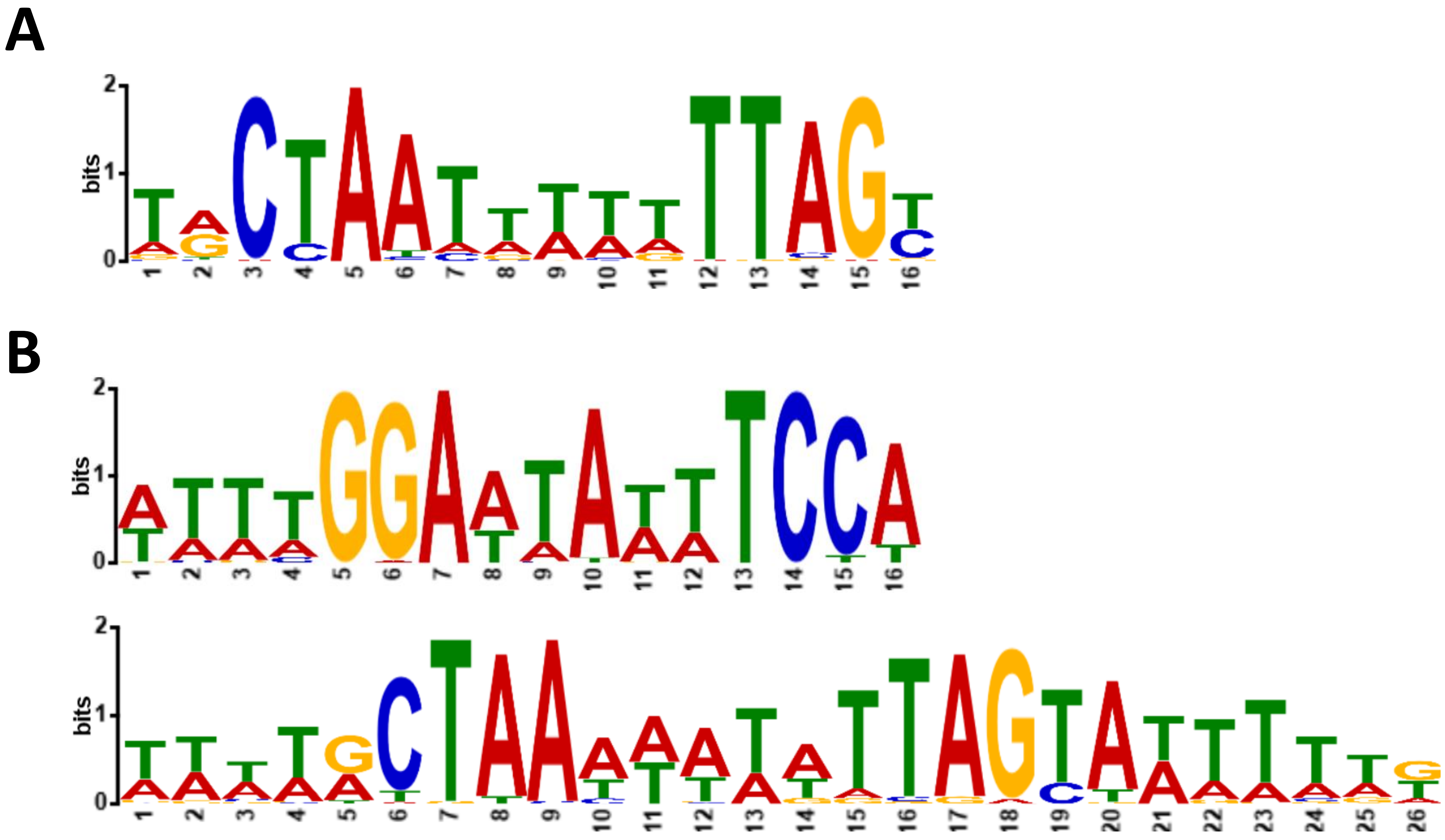

Figure S2 - Results for MEME motif discovery without constraint for palindromic motifs on Bacteroidetes promoter sequences encoding UmuD (A) and DinB/UmuC (B) proteins.
