# Supplementary material for: Non-canonical LexA proteins regulate the SOS response in the Bacteroidetes
Source: Nucleic Acids Res. 2021 Oct 6;49(19):11050–66. doi: 10.1093/nar/gkab773 (PMC8565304; doi:10.1093/nar/gkab773)
Supplement: gkab773_Supplemental_Files [file gkab773_supplemental_files.zip › Supplementary Figure 5.pdf]

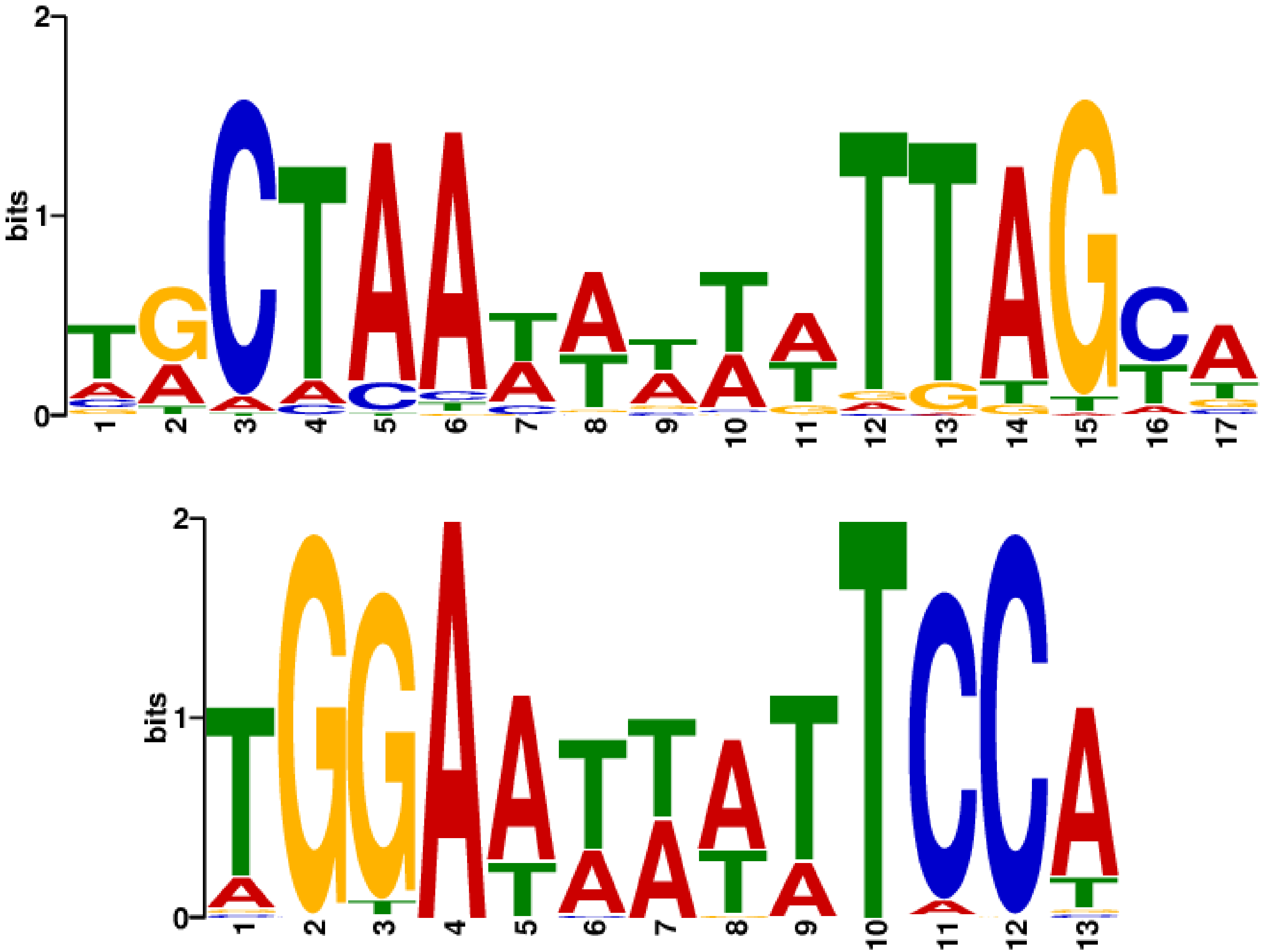

Figure S5 - Results for MEME motif discovery on the promoter sequences of all identified genes encoding putative Bacteroidetes SOS regulators (Supplementary Table S8).
