# Supplementary material for: Non-canonical LexA proteins regulate the SOS response in the Bacteroidetes
Source: Nucleic Acids Res. 2021 Oct 6;49(19):11050–66. doi: 10.1093/nar/gkab773 (PMC8565304; doi:10.1093/nar/gkab773)
Supplement: gkab773_Supplemental_Files [file gkab773_supplemental_files.zip › Supplementary Figure 6.pdf]

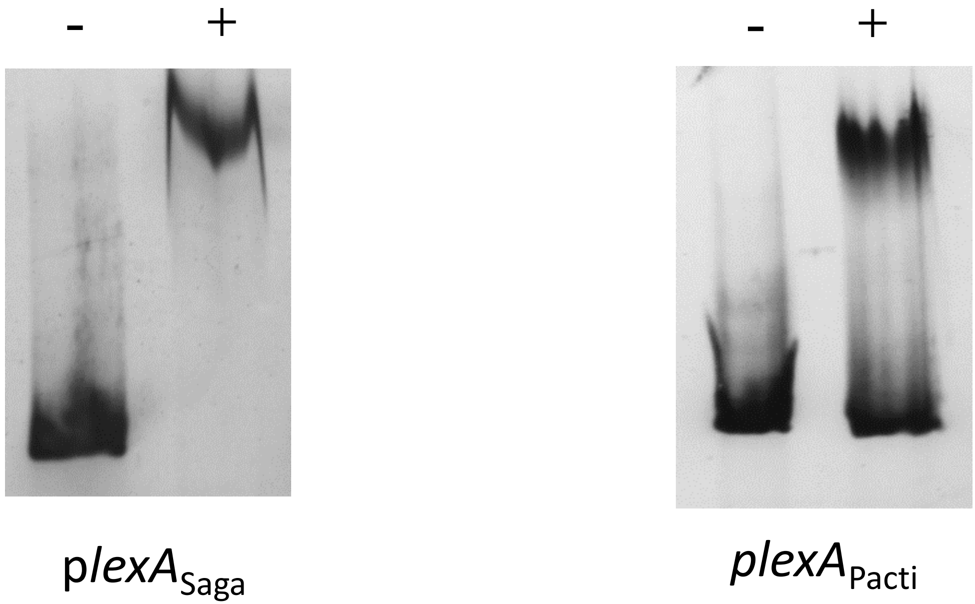

Figure S6 - Electromobility-shift assays with purified *S. agarivorans* (Saga) and *P. actiniarum* (Pacti) SOS regulators against the promoters of the genes encoding them. The “-” symbol denotes absence of protein and “**+**” the presence of protein in the mixture.
