# Supplementary material for: Non-canonical LexA proteins regulate the SOS response in the Bacteroidetes
Source: Nucleic Acids Res. 2021 Oct 6;49(19):11050–66. doi: 10.1093/nar/gkab773 (PMC8565304; doi:10.1093/nar/gkab773)
Supplement: gkab773_Supplemental_Files [file gkab773_supplemental_files.zip › Supplementary material captions-.pdf]

Supplementary material captions

Data S1 - JSON-formatted input file for CGB comparative genomics analyses using the GGA-N5-TCC motif.

Data S2 - JSON-formatted input file for CGB comparative genomics analyses using the CTAA-N5-TTAG motif.

Data S3 - Promoter sequences used for motif discovery on genes encoding Bacteroidetes UmuD homologs.

Data S4 - Promoter sequences used for motif discovery on genes encoding Bacteroidetes DinB/UmuC homologs.

Data S5 - FASTA-formatted multiple sequence alignment of the C-terminal regions of Bacteroidetes SOS repressors and S24 family proteins with experimentally-reported C-terminal domain structure.

Data S6 - Nexus-formatted file for the Bacteroidetes SOS regulators and COG1974 and COG2932 representatives phylogenetic tree.

Data S7 - PDB-formatted files with the Robetta predicted structures for the putative Bacteroidetes SOS regulators *S. agarivorans* DSM 23515 [WP_075324850.1] and *P. actiniarum* DSM 19842 [WP_025606010.1] and their trimmed C- and N-terminal domains.

Data S8 - Nexus-formatted file for Bacteroidetes RecA proteins phylogenetic tree.

Table S1 - List of oligonucleotides used in this work.

Table S2 - List of reference experimentally-validated LexA proteins. For each protein, the species name, protein accession and source are provided.

Table S3 - List of BLASTP hits with *E. coli* UmuC and DinP proteins against Bacteroidetes complete genomes.

Table S4 - HMMER hmmsearch results for COG1974 and COG0389 HMM models against complete Bacteroidetes proteomes.

Table S5 - List of SOS genes with two or more predicted binding sites for the motifs targeted by putative Bacteroidetes SOS regulators.

Table S6 - List of UmuD- and DinB-encoding genes in each of the complete Bacteroidetes proteomes analyzed.

Table S7 - HHMER hmmsearch results for the PF01381 HMM model against sequences of putative Bacteroidetes SOS regulators.

Table S8 - Predicted binding sites following the GGA-N5-TCC and CTAA-N5-TTAG motifs upstream of the genes encoding the putative Bacteroidetes SOS regulators.

Table S9 - Homologs of the putative Bacteroidetes SOS regulators identified with BLASTP.

Table S10 - Perfect dyad motifs with 5 bp spacer identified in the promoter sequences of Bacteroidetes SOS regulators lacking an instance of the CTAA-N5-TTAG motif. For each regulator, the table reports the species, genome accession number, gene locus tag, sequence of the identified dyad motif and distance to the predicted translational start site for the SOS regulator gene.

Figure S1 - Flow diagram of the computational pipeline used in this work. (1) Hidden Markov Models for reference COGs are used to search reference genomes with hmmsearch in order to detect orthologs of genes likely to be regulated by the SOS response. (2) Biopython scripts are used to retrieve the corresponding promoter sequences and filter them by sequence similarity to generate a library of diverse promoters on which to perform motif discovery. (3) Motif discovery is performed on the promoter library using MEME. (4) Inferred motifs are used to elucidate putative regulatory networks using the CGB comparative genomics suite. (5) Binding of putative regulators and DNA damage induction are validated for target genes with identified sites for inferred motifs. This figure was constructed using some BioRender templates.

Figure S2 - Results for MEME motif discovery without constraint for palindromic motifs on Bacteroidetes promoter sequences encoding UmuD (A) and DinB/UmuC (B) proteins.

Figure S3 - CGB-generated full heatmap showing the posterior probability of regulation (green) for orthologous groups in Bacteroidetes species where a GGA-N5-TCC motif instance has been detected.

Figure S4 - CGB-generated full heatmap showing the posterior probability of regulation (green) for orthologous groups in Bacteroidetes species where a CTAA-N5-TTAG motif instance has been detected.

Figure S5 - Results for MEME motif discovery on the promoter sequences of all identified genes encoding putative Bacteroidetes SOS regulators (Table S8).

Figure S6 - Electromobility-shift assays with purified *S. agarivorans* and *P. actiniarum* SOS regulators against the promoters of the genes encoding them. The “-” symbol denotes absence of protein and “+” the presence of protein in the mixture.
