# Supplementary figures and images for: Non-canonical LexA proteins regulate the SOS response in the Bacteroidetes
Source: Nucleic Acids Res. 2021 Oct 6;49(19):11050–66. doi: 10.1093/nar/gkab773 (PMC8565304; doi:10.1093/nar/gkab773)

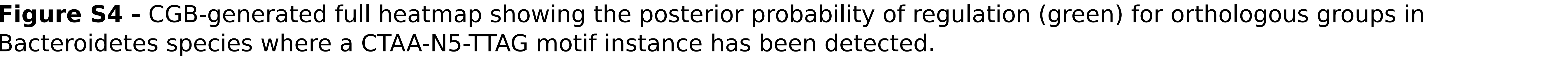

Supplement: gkab773_Supplemental_Files [file gkab773_supplemental_files.zip › Supplementary Figure 4.pdf]
